# Con‐Ca^2+^‐tenating plant immune responses via calcium‐permeable cation channels

**DOI:** 10.1111/nph.18044

**Published:** 2022-03-17

**Authors:** Nak Hyun Kim, Pierre Jacob, Jeffery L. Dangl

**Affiliations:** ^1^ Department of Biology and Howard Hughes Medical Institute University of North Carolina at Chapel Hill Chapel Hill NC 27599 USA

**Keywords:** calcium‐permeable cation channel, disease resistance, effector‐triggered immunity, NLR immune receptor, pattern recognition receptor, pattern‐triggered immunity

## Abstract

Calcium serves as a second messenger in a variety of developmental and physiological processes and has long been identified as important for plant immune responses. We discuss recent discoveries regarding plant immune‐related calcium‐permeable channels and how the two intertwined branches of the plant immune system are intricately linked to one another through calcium signalling. Cell surface immune receptors carefully tap the immense calcium gradient that exists between apoplast and cytoplasm in a short burst via tightly regulated plasma membrane (PM)‐resident cation channels. Intracellular immune receptors form atypical calcium‐permeable cation channels at the PM and mediate a prolonged calcium influx, overcoming the deleterious influence of pathogen effectors and enhancing plant immune responses.

## Abstract 2

See also the Commentary on this article by Lee & Romeis, **234**: 769–772.

1

**Table:** 

	Contents
	Summary	813
I.	Introduction	813
II.	Regulation of calcium levels under normal conditions	814
III.	PTI and ETI converge over Ca^2+^ regulation	814
IV.	Regulation of calcium levels by PRR signalling	814
V.	Regulation of calcium levels by NLR signalling	816
VI.	Translating cytosolic calcium to defence signalling	816
VII.	Conclusions	817
	Acknowledgements	817
	References	817

## I. Introduction

Plants use plasma membrane (PM) localised receptors to recognise extracellular pathogen‐associated molecular patterns (PAMPs) and trigger PAMP‐triggered immunity (PTI). PAMP‐triggered immunity is characterised by a transient immune response that is sufficient to restrict the growth of nonadapted microbes. Virulent pathogens, however, have evolved virulence effectors that are capable of modulating PTI, rendering it inefficient. Plants counteract the effector disruptions of PTI with an arsenal of intracellular sensor nucleotide binding‐leucine rich (NB‐LRR) receptors (NLRs) that recognise pathogen effectors and boost the immune response to effectively bypass the effector‐blocked PTI response (Jones & Dangl, 2006). Although PTI and effector‐triggered immunity (ETI) are initiated by different receptors, pattern recognition receptor (PRR) and NLR signalling potentiate a largely overlapping defence response but with different amplitudes (Tian *et al*., 2021; Yuan *et al*., 2021). ‘Sensor’ NLRs possess either a N‐terminal coiled‐coil (CC) domain or Toll‐interleukin‐1 receptor/resistance (TIR) domains. Coiled‐coil‐domain NLRs (CNLs) are functionally self sufficient and TIR‐domain NLRs (TNLs) require ‘helper’ CC^RPW8^‐containing NLRs (RNLs) to function (Jubic *et al*., 2019). Effector‐triggered immunity mediated by NLRs is a high amplitude and lasting immune response that often culminates in the death of the host cell (Jones *et al*., 2016). Recent studies have revealed the molecular function of at least some CNLs and RNLs as calcium‐permeable cation channels (Bi *et al*., 2021; Jacob *et al*., 2021). This discovery highlights the importance of Ca^2+^ in defence and allows us to describe plant immune signalling from the perspective of the receptor's molecular function.

## II. Regulation of calcium levels under normal conditions

**Fig. 1:**
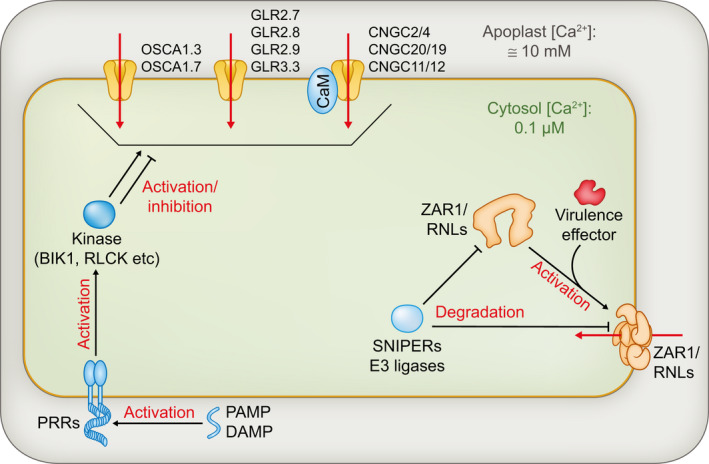
The modulation of cytoplasmic calcium levels during the plant immune response. Overview of the calcium channels/calcium‐permeable channels involved in calcium homeostasis during plant immunity. Pattern recognition receptor (PRR) signalling regulates the activity of plasma membrane‐resident channels, directly or indirectly through phosphorylation. By contrast, NB‐LRR receptors (NLRs) are regulated at the level of protein abundance, notably by SNIPER E3 ligases, and form a cation channel only after activation. Red arrows indicate Ca^2+^ flux. Blunt‐ended arrows indicate inhibition. BIK1, botrytis‐induced kinase 1; CaM, calmodulin; CNGC, cyclic nucleotide‐gated channel; DAMP, damage‐associated molecular pattern; GLR, glutamate receptor‐like; OSCA, reduced hyperosmolality, induced Ca^2+^ increase; PAMP, pathogen‐associated molecular pattern; RLCK, receptor like cytoplasmic kinase; RNL, CCRPW8‐NLR; SNIPER, snc1‐influencing plant E3 ligase reverse; ZAR1, HOPZ‐activated resistance 1.

Ca^2+^ is an essential nutrient that also acts as a potent secondary messenger for all aspects of plant physiology including development, abiotic stress and defence (Luan & Wang, 2021). Under normal, nonstimulating conditions, free cytosolic Ca^2+^ levels are kept at a low level (around 0.1 µM), which prevents Ca^2+^ cytotoxicity and enables minute Ca^2+^ level changes to act as signals (Thor, 2019). Low cytoplasmic Ca^2+^ levels are maintained via Ca^2+^ export to the apoplast and sequestration into the vacuole or chloroplast by H^+^/Ca^2+^ antiporters and Ca^2+^‐ATPases that results in an immense buildup of Ca^2+^ (up to 10 mM) in apoplast and organelles (Thor, 2019; Hilleary *et al*., 2020). Upon activation, Ca^2+^ channels produce specific Ca^2+^ signatures defined by the frequency and amplitude of the Ca^2+^ level variation they support. The Ca^2+^ signature determines which Ca^2+^‐regulated factors will respond to the stimulus and for how long (Whalley & Knight, 2013). The regulation of cytoplasmic Ca^2+^ levels during the plant immune response involves a large number of channels (please refer to Fig. 1).

## III. PTI and ETI converge on cytosolic Ca^2+^ regulation

PAMP‐triggered immunity is typically initiated by ligand recognition and a consequent phosphorylation cascade. Pattern recognition receptors (PRRs) are either kinases themselves, receptor‐like kinases (RLKs) that function directly with RLK or RLP co‐receptors and/or function with receptor‐like cytoplasmic kinases, receptor‐like proteins (RLPs) (Macho & Zipfel, 2014). Pattern recognition receptor‐initiated phosphorylation cascades regulate the activity of plasma membrane‐resident Ca^2+^ channels that trigger rapid Ca^2+^ bursts and the transient activation of a mitogen‐activated protein kinase (MAPK) cascade (Tian *et al*., 2019; Yu *et al*., 2019; Thor *et al*., 2020). Effector‐triggered immunity responses mediated by the sensor CNL HOPZ‐ACTIVATED RESISTANCE 1 (ZAR1) or helper RNLs trigger the formation of Ca^2+^ permeable channels that consist of structured oligomers of the NLR receptors themselves (Wang *et al*., 2019; Bi *et al*., 2021; Jacob *et al*., 2021). This results in a lasting Ca^2+^ influx and sustained activation of the MAPK cascade, possibly through the action of calcium‐dependent protein kinases (CDPKs or CPKs) (Bredow & Monaghan, 2019). Constitutive increases in either cytoplasmic Ca^2+^ levels or MAPK activity are sufficient to activate a strong immune response and ectopic cell death (Yoshioka *et al*., 2006; Genot *et al*., 2017; Hilleary *et al*., 2020; Zhao *et al*., 2021). However, in the absence of a PRR‐triggered kinase cascade, Ca^2+^ influx resulting from transient NLR activation is not sufficient to trigger defence and cell death (Ngou *et al*., 2021; Yuan *et al*., 2021). This suggests that, whereas PRR‐induced phosphorylation cascade and transient Ca^2+^ burst activate an immune response, sustained NLR‐driven Ca^2+^ influx heightens its intensity (Fig. 1).

## IV. Regulation of calcium levels by PRR signalling

**Fig. 2:**
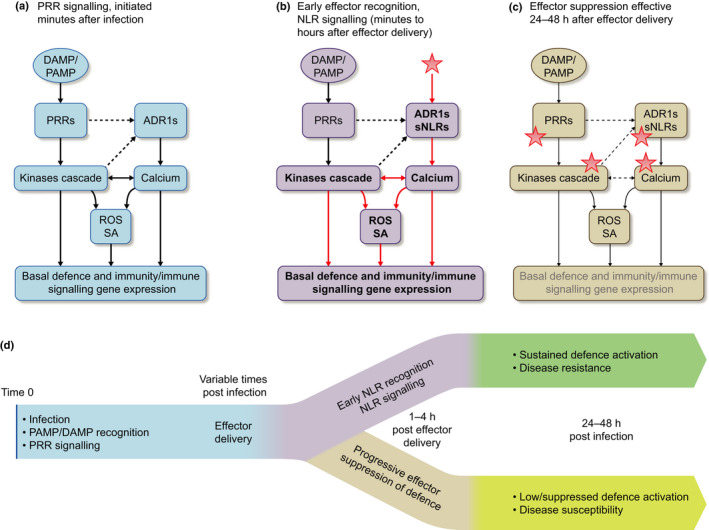
Pattern recognition receptor (PRR) and NB‐LRR receptor (NLR) signalling cooperatively regulates defence activation. (a) Pathogen infection is recognised at the plasma membrane by PRRs. Within minutes, PRRs trigger a phosphorylation cascade (involving receptor‐like cytoplasmic kinases (RLCKs) and mitogen‐activated protein kinases (MAPKs)) that activates Ca^2+^ channels, leading to a transient Ca^2+^ burst. Kinases and Ca^2+^‐responsive proteins then synergistically induce the accumulation of reactive oxygen species (ROS) and salicylic acid (SA), defence gene expression and immune signalling gene expression. The ‘helper’ NLR ACTIVATED DISEASE RESISTANCE 1 (ADR1) may participate to the PRR‐triggered Ca^2+^ influx by directly forming Ca^2+^‐permeable channels in the plasma membrane, but the mechanism linking PRRs to helper NLRs is still unknown (dotted arrows). (b) Generally, a few hours after infection, virulent pathogens deploy effectors (red stars) to modulate the plant immune system. If an intracellular ‘sensor’ NLR (sNLR) can recognise one of these effectors, it will become active and trigger, directly or indirectly, a strong and long‐lasting cytosolic Ca^2+^ influx. Elevated Ca^2+^ levels, together with PRR signalling, will lead to strong defence activation and disease resistance, a process known as effector‐triggered immunity (ETI). (c) In the absence of NLR recognition, pathogen effectors will progressively dampen the plant immune system and trigger disease susceptibility. (d) Overview of the activation of PRR signalling, NLR signalling and effector delivery, with time, during infection with a virulent pathogen with or without effector recognition. Red arrows indicate ETI‐specific signalling. Arrow width refers to signalling intensity. ADR1, ACTIVATED DISEASE RESISTANCE 1; DAMP, damage‐associated molecular pattern; PAMP, pathogen‐associated molecular pattern.

Cation channels belonging to the glutamate receptor‐like (GLR), the reduced hyperosmolality induced Ca^2+^ increases (OSCAs) and the cyclic nucleotide‐gated channels (CNGCs) families are involved in PRR signalling (Tian *et al*., 2019) (Fig. 2a). Loss of function of CNGC2 or CNGC4 triggers the so‐called ‘defence no death’ (DND) phenotype characterised by the constitutive activation of defence and the loss of hypersensitive cell death (Clough *et al*., 2000). CNGC2 and CNGC4 form heteromeric channels that are important for proper Ca^2+^ nutrition and for preventing overaccumulation of Ca^2+^ in the apoplast and the cell wall (Wang *et al*., 2017; Tian *et al*., 2019). Under low extracellular Ca^2+^ conditions, the defence no death phenotype is reverted (Tian *et al*., 2019). Therefore, the CNGC2/4 heteromer is not required for NLR signalling. Rather, in *dnd* mutants, elevated extracellular Ca^2+^ levels heighten defence activation and inhibit cell death through an unknown mechanism that may involve SA (Zavaliev *et al*., 2020).

However, biochemical evidence links CNGC2/4 to PRR signalling. CNGC2/4 is activated by the receptor‐like cytoplasmic kinase (RLCK) *Botrytis*‐induced kinase 1 (BIK1) and is required for PAMP‐induced Ca^2+^ burst and defence against the effector delivery‐deprived *Pst* DC3000 Δ*HrcC* (Tian *et al*., 2019). Like the defence no death phenotype, the requirement for CNGC2/4 in PRR signalling is suppressed under low Ca^2+^ conditions. Perhaps overaccumulation of extracellular calcium leads to the inhibition or degradation of Ca^2+^ channels that would otherwise compensate for the loss of CNGC2/4 in PRR signalling. Consistent with this hypothesis, the activity of CNGCs themselves is tightly regulated by Ca^2+^ levels via calmodulins (CaMs) (DeFalco *et al*., 2016; Dietrich *et al*., 2020). In addition, CNGCs can be regulated at the level of protein abundance. CNGC20 forms a heteromeric channel with CNGC19 that is regulated by PRR signalling and required for defence against *Piriformospora indica* (Yu *et al*., 2019; Jogawat *et al*., 2020). CNGC20 abundance is negatively regulated by the PRR co‐receptor BRI1‐ASSOCIATED RECEPTOR KINASE (BAK1) under normal conditions. In the absence of BAK1, CNGC20 overaccumulates and triggers ectopic defence and cell death. (Fig. 1).

Redundancy of PRR‐regulated Ca^2+^ channels is important. CNGC2/4 and CNGC20/19 only contribute a fraction of the PRR‐triggered Ca^2+^ influx (Tian *et al*., 2019; Jogawat *et al*., 2020). The GLR cation channels are also important contributors to the PRR‐induced calcium spike as evidenced by the use of GLR inhibitors and loss‐of‐function mutants (Manzoor *et al*., 2013). GLR3.3 is required for half of oligogalacturonide‐triggered calcium influx (Manzoor *et al*., 2013). The triple mutant *glr2.7 glr2.8 glr2.9* showed a very slight but significant reduction in three different PRR‐associated calcium bursts (Bjornson *et al*., 2021). Some channels are cell‐type specific, as the BIK1‐regulated Ca^2+^ channels OSCA1.3 and OSCA1.7, which significantly contribute to the PAMP‐triggered Ca^2+^ influx in guard cells but do not impact mesophyll cell responses (Thor *et al*., 2020). Although they are required for stomatal closure, the *osca1.3 osca1.7* double mutant still retains some Ca^2+^ influx upon PAMP treatment indicating that other channels are involved in stomatal defence.

## V. Regulation of calcium levels by NLR signalling

Diverse virulence effectors inhibit PRR‐mediated Ca^2+^ influx during infection with virulent pathogens (Lammertz *et al*., 2019). Recently, the CNL ZAR1 and the RNLs ACTIVATED DISEASE RESISTANCE 1 (ADR1) and N REQUIREMENT GENE 1.1 (NRG1.1) were shown to encode Ca^2+^‐permeable channels enriched at the PM (Bi *et al*., 2021; Jacob *et al*., 2021). Both ZAR1 and RNLs were shown to transport Ca^2+^ and other cations via electrophysiology in artificial membrane (ZAR1) and human HEK293 cells (RNLs), in the absence of any putative plant ion channels partners. They were both found to be impermeable to chloride anions or larger molecules such as tetraethylammonium ions, suggesting they form a narrow and cation‐specific channels (Bi *et al*., 2021; Jacob *et al*., 2021). Other CNLs like RPS2 and RPM1 trigger a long‐lasting Ca^2+^ influx that is required for defence and cell death (Grant *et al*., 2000; Gao *et al*., 2013; Jubic *et al*., 2019). The direct promotion of cytosolic Ca^2+^ influx by NLRs provides a new mechanism, independent of PRR‐mediated calcium influx, required for NLR‐mediated cell death responses in the presence of effectors (Fig. 2b).

ZAR1 and RNLs are not typical Ca^2+^ channels. In the resting state, ZAR1 and RNLs are absent from the PM and activation leads to channel assembly and PM insertion. The fact that the NLR‐driven calcium influx is long lasting and can lead to cell death suggests that NLR channel activities may not be under Ca^2+^/CaM regulation as found for the CNGCs (Tian *et al*., 2019; Bi *et al*., 2021; Jacob *et al*., 2021). In fact, NLRs are apparently only regulated at the channel assembly level, in response to effectors, and by protein turnover. Indeed, the SNIPER1 and SNIPER2 E3 ligases regulate broadly NLR protein levels by targeting the shared NB domains of sensor NLRs (Wu *et al*., 2020). This differential mode of regulation may explain why NLRs, in contrast with RR‐regulated channels, overcome effector suppression of PTI and trigger cell death.

Pattern recognition receptor and NLR signalling may cooperate to regulate calcium levels. CNGC11/12 are apparently not required for PRR signalling but do contribute to resistance to either *Hyaloperonospora arabidopsidis* Emwa1 or *Pseudomonas syringae* DC3000(*avrRpt2*), which both trigger specific NLRs (Yoshioka *et al*., 2006; Moeder *et al*., 2011). However, CNGC11/12 are negatively regulated by CaM in the presence of high calcium levels, which is inconsistent with the steady and lasting elevation of cytosolic calcium levels during NLR signalling. Also, CNGC11/12 are not required for cell death induction during NLR signalling, so it is unclear if CNGC11/12 contribute to calcium influx during NLR signalling or potentiate NLR activation in the context of PRR signalling (Ngou *et al*., 2021; Yuan *et al*., 2021). By contrast, NLRs are involved in signalling and defence priming triggered by some PRRs. RNLs of the ADR1 family are involved in some PRR signalling and are required for some PRR‐driven defence priming (Pruitt *et al*., 2021; Tian *et al*., 2021). It remains unknown how ADR1s are activated by PRR signalling and how they impact cytosolic Ca^2+^ levels during PRR signalling.

## VI. Translating cytosolic calcium to defence signalling

Various calcium‐binding proteins such as CaM, CAM‐like proteins (CMLs), calcineurin B‐like proteins (CBLs), Ca^2+^‐dependent protein kinases (CDPKs or CPKs), phospholipase D and respiratory burst oxidase homologue (RBOH) NADPH oxidases, among others, translate cytoplasm Ca^2+^ levels into downstream transcriptional defence signals (Luan & Wang, 2021). CaM‐binding transcription activator (CAMTA) 3 acts as a mediator of convergent transcriptional regulation from NLR‐ and PRR‐mediated signalling, as demonstrated by a dominant‐interfering *camta3* mutant that exhibited compromised PTI‐ and ETI‐mediated transcriptional responses in Arabidopsis (Jacob *et al*., 2018). CAMTA3 directly binds to promoters of CALMODULIN‐BINDING PROTEIN 60g (CBP60g) and SYSTEMIC ACQUIRED RESISTANCE DEFICIENT1 (SARD1) and loss of *CAMTA3* leads to constitutive activation of immune responses suggesting its function as transcriptional repressors (Sun *et al*., 2020). CAMTA3 appears to be targeted by virulence effectors and is guarded by two TNLs, DSC1 and DSC2 (Lolle *et al*., 2017). Defence upregulation in *camta* mutants is thus induced by constitutive NLR activation.

CBP60 family transcription factors include both positive and negative immune regulators (Li *et al*., 2021). CBP60a is known as a negative regulator while positive regulators like CBP60b or CBP60g bind and activate promoters of defence genes such as isochorismate synthase 1 (ICS1), an essential enzyme for synthesis of SA (Li *et al*., 2021), which induces the expression of pathogenesis‐related genes and plays critical roles in plant immunity (Zhang & Li, 2019). CBP60b is also likely a guardee of the TNL SNC1, like CAMTAs illustrating the importance of Ca^2+^‐responsive factors in defence (Li *et al*., 2021).

Arabidopsis CBL1 and CBL9 recruit CBL‐interacting protein kinase (CIPK) 26 to the PM where it phosphorylates NADPH oxidase. This produces reactive oxygen species (ROS) that may be toxic to invading pathogens and can act as potent defence signalling molecules (Ma *et al*., 2020). By contrast, CIPK6 has been shown to negatively regulate ROS production in PTI and ETI (Ma *et al*., 2020). CPK1/2/4/11 have also been shown to activate ROS signalling by phosphorylating NADPH oxidases (Gao *et al*., 2013). In addition to ROS, direct phosphorylation of a specific subgroup of defence‐related WRKY transcription factors has been reported upon sustained activation of Arabidopsis CPK4/5/6/11 (Gao *et al*., 2013). Calcium‐dependent protein kinases also connect calcium signalling to MAPK pathways, as they are capable of phosphorylating MAP kinases (Bredow & Monaghan, 2019).

How Ca^2+^ regulates CNL‐ and RNL‐mediated cell death is not fully understood. Due to its cytotoxicity, Ca^2+^ overaccumulation could be directly disrupting cellular homeostasis and triggering cell death. Alternatively, cell death could result from NLR‐mediated pore formation, leading to the activation of Ca^2+^‐responsive proteins that would specifically respond to the precise Ca^2+^ signatures. These are not mutually exclusive. Cell death and defence can be dissociated during NLR signalling (so‐called ‘extreme resistance’; Ross *et al*., 2021) and some CPKs contribute to cell death induction, arguing for an active cell death process (Gao *et al*., 2013; Laflamme *et al*., 2020).

Overall, intricate networks of diverse calcium‐binding proteins translate elevated cytosolic Ca^2+^ levels into transcriptional reprogramming through MAPKs, WRKY, CAMTA and CBP60 transcription factors (Gao *et al*., 2013; Bredow & Monaghan, 2019). Simultaneous activation of SA and ROS signalling pathways fine‐tunes the transcription machinery toward defence responses, rather than other Ca^2+^‐dependent processes, putatively via antagonistic actions of SA (Moeder *et al*., 2010). The exact nature of the defence response may differ depending on the immediate composition of the calcium decoders. By regulating the expression of the calcium‐binding proteins, PTI potentiates and shapes ETI and vice versa (Ngou *et al*., 2021; Yuan *et al*., 2021). We currently lack the precise understanding of the calcium signatures generated by NLRs and whether individual NLRs generate unique signatures that determine the eventual outcome, or, alternatively, whether the outcome depends on the downstream calcium decoders.

## VII. Conclusions

The discovery that ZAR1 and RNLs are calcium‐permeable cation channels significantly altered our understanding of the intricate relationship between the CNL and TNL branches of NLR immune receptor function. Although PRR and NLR signalling both converge on the cytoplasmic Ca^2+^ influx, the PRR‐driven Ca^2+^ influx is tightly regulated by phosphorylation and CaM or related proteins. Pattern recognition receptor signalling is essentially transient, which is important to avoid the negative impact of immune system activation on growth. The distinct long‐lasting calcium influx generated by NLRs suggests a much lower or even a total lack of calcium‐dependent negative feedback on the channel activity (Grant *et al*., 2000; Bi *et al*., 2021; Jacob *et al*., 2021). This differential regulation may explain why NLR signalling cannot be dampened by the pathogen virulence effectors that inhibit PRR signalling, even though both PRRs and NLRs signal through largely similar downstream signal transduction cascades. Alternatively, as PRR signalling is required together with NLR signalling to trigger strong immunity (Yuan *et al*., 2021), it should be considered that NLR signalling overcomes effector action by boosting defence before effectors can effectively suppress it (Fig. 2). The initial PRR signalling activates a fast and transient phosphorylation cascade and Ca^2+^ burst, starting a few minutes after PAMP recognition (Fig. 2a). Together, these events orchestrate the transient accumulation of ROS, SA and defence molecules, among which are Ca^2+^‐responsive transcription factors. Over the first few hours following infection, depending on the pathogen, virulent pathogens inject intracellular effectors that dampen PRR signalling. After delivery, effectors render the cell refractory to PAMP stimulation (Fig. 2c), but at early time points 1–4 h after infection, PRR signalling is still actively upregulating defence‐related genes that include Ca^2+^‐responsive elements (Fig. 2b). In this primed state, effector‐activated NLRs can outpace or bypass effector‐suppressed PRR signalling by delivering overwhelming Ca^2+^, which rapidly leads to NLR‐mediated cell death and, likely, ETI.

## Author contributions

NHK and PJ contributed equally to this work.
